# A microfibre assembly of an iron-carbon composite with giant magnetisation

**DOI:** 10.1038/srep03051

**Published:** 2013-10-29

**Authors:** Ying Liang, Pu Liu, Jun Xiao, Hongbo Li, Chengxin Wang, Guowei Yang

**Affiliations:** 1 State Key Laboratory of Optoelectronic Materials and Technologies, Institute of Optoelectronic and Functional Composite Materials, Nanotechnology Research Center, School of Physics & Engineering, Sun Yat-sen University, Guangzhou 510275, Guangdong, P. R. China; 2 These authors contributed equally to this work.

## Abstract

Iron carbide is among the oldest known materials. The utility of this ancient advanced material is greatly extended in its nanostructured forms. We demonstrate for the first time that one-dimensional iron carbide microfibres can be assembled in liquid using strong magnetic field-assisted laser ablation. The giant saturation magnetisation of these particles was measured a 261 emu/g at room temperature, which is the best value reported to date for iron nitride and carbide nanostructures, is 5.5 times greater than the 47 emu/g reported for Fe_3_C nanoparticles, and exceeds the 212 emu/g for bulk Fe. The magnetic field-induced dipolar interactions of the magnetic nanospheres and the nanochains played a key role in determining the shape of the product. These findings lead to a variety of promising applications for this unique nanostructure including its use as a magnetically guided transporter for biomedicine and as a magnetic recording material.

Interest in the self-assembly of magnetic nanoparticles (MNPs) into larger ordered structures as an approach for engineering new materials and devices^1^ such as photonic crystals^2^, magnetotransporters^3^, micromechanical sensors^4^, magnetic memory materials^5^, magnetic switching devices^6^, magnetic force probes^7^, and DNA separation materials^8^ among others^9^,^10^ has recently increased. Therefore, developing generally applicable and effective techniques for the assembly of ordered nanostructures of MNPs is essential. For nanochain or chain-like MNP nanostructures, achieving an anisotropic one-dimensional (1D) assembly from isotropic, zero-dimensional MNPs remains challenging^11^,^12^. Therefore, an effective and repeatable assembly of 1D MNP chains is a significant step toward realising their potential as new materials in practical applications. A number of versatile routes have been established to produce a variety of 1D MNP chains using either direct interactions (e.g., interparticle forces) or indirect methods requiring a template or an external field^12^,^13^,^14^,^15^,^16^. The use of external magnetic fields to induce the MNP assembly is simple, clean, and inexpensive^17^. We developed a unique and facile approach for assembling the 1D MNP chains using magnetic field-assisted laser ablation in liquid (MF-LAL). Using this technique, we assembled micro-fibres of submicron iron carbide spheres and characterised their magnetic properties. The assembled microfibres were ferromagnetic, with giant magnetisations of 261 and 295 emu/g at 300 K and 5 K, respectively, which are the best values reported to date for iron nitride and carbide nanostructures^18^. Additionally, MF-LAL is simple, green, and catalyst-free allowing researchers to choose and design interesting solid targets and solution environments to fabricate ordered magnetic nanostructures for fundamental research and potential applications.

## Results

### Structure and morphology of the as-assembled microfibres

**Figure 1:**
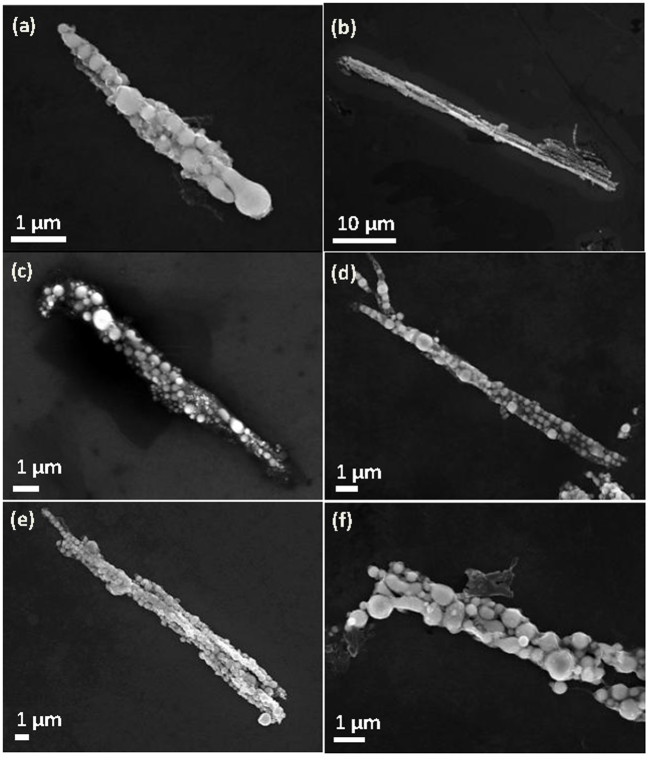
SEM images of the micro-fibers under different experimental conditions. (a) 1 T, 100 mJ and 5 Hz; (b) 5 T, 100 mJ and 5 Hz; (c) 2 Hz, 9 T and 100 mJ; (d) 10 Hz, 9 T and 100 mJ; (e) 50 mJ, 9 T and 5 Hz; (f) 150 mJ, 9 T and 5 Hz.

**Figure 2:**
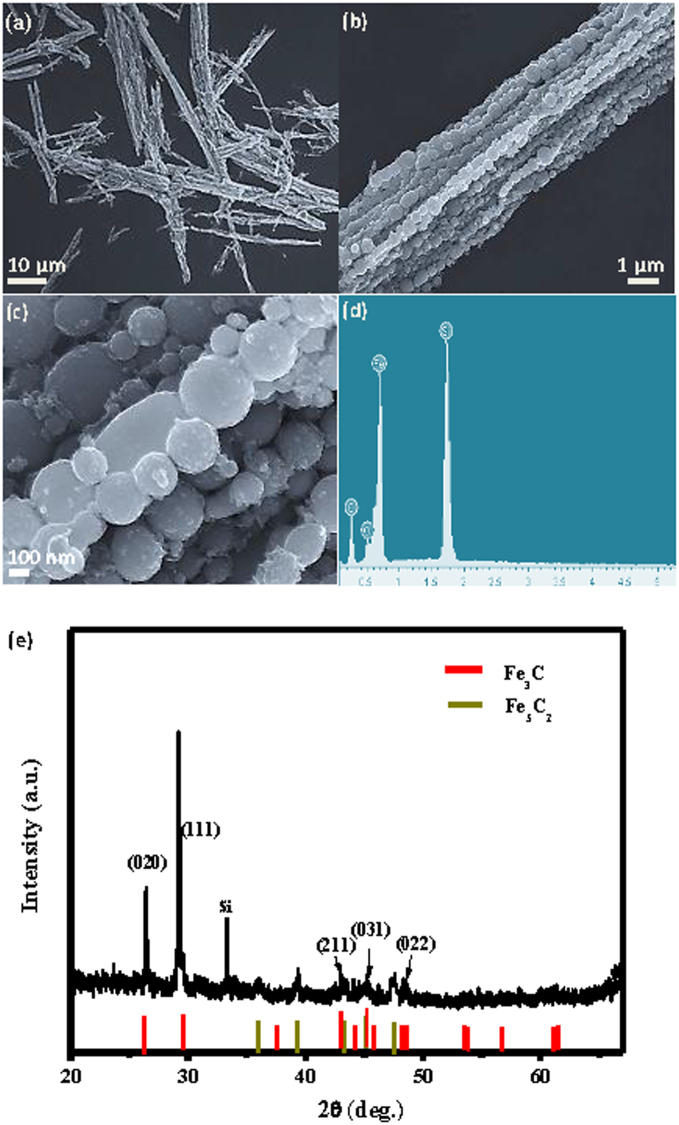
SEM images of the micro-fibers fabricated at 9 T, 100 mJ and 5 Hz (a–c), and corresponding EDS (d) and XRD patterns (e) for the products.

This study systematically examines the influence of the important fabrication parameters including the magnetic field, the repeating frequency, and the pulse energy of the pulsed-laser. Typical microfibres were fabricated under various conditions. Figure 1a–b demonstrates the influence of the magnetic field intensity on the fabrication when all other experimental conditions are held constant. Under a reduced magnetic field (1 T in Fig. 1a), the microfibres are short and bent compared with those under a higher magnetic field (5 T in Fig. 1b), which indicates that a strong magnetic field favours the fabrication of long, straight microfibres. Similarly, Figure 1c–d demonstrates the influence of the repeating laser frequency on the fabrication of the microfibres. We observed no significant difference between 2 Hz (Fig. 1c) and 10 Hz (Fig. 1d). However, the laser ablation in ethanol at the higher repeating frequency results in the undesirable burning of ethanol. A low repeating frequency reduces the product yield and further affects the formation of the microfibres. Thus, we hypothesise that a median repeating frequency is preferable. Moreover, a low energy is likely desirable for the formation of small and uniform-sized nanoparticles as shown in Fig. 1e (50 mJ) and f (150 mJ). However, the size of the nanoparticles can also be largely affected by the laser irradiation during the ablation process. Based on our systemic analysis, we can conclude that that a magnetic field of 9 T, a pulse repeating frequency of 5 Hz, and a pulse repeating energy of 100 mJ are the optimal experimental parameters for assembling the microfibre chain via MF-LAL. The products assembled under these conditions are presented in Fig. 2a–e and were several tens of micrometres long (~20–60 μm) and several micrometres in diameter. These ordered microfibres were constructed from 1D chains of spheres tightly bundled in an orderly fashion as depicted in Fig. 2b. We thus deduced that these microstructures consisted of linear, bunched chains of interconnected spherical particles. Spherical nanoparticles are often produced using LAL^19^. The average size of the as-synthesised spheres was approximately 200–300 nm, as shown in Fig. 2c. Careful observations revealed some nanospheres with diameters of ~10–20 nm on the surfaces of the nanochains. Some of the nanospheres were stretched along the chain direction induced by the magnetic field. The corresponding energy-dispersive X-ray spectrometer (EDS) pattern (Fig. 2d) indicated that the microfibres were primarily composed of Fe, C, O, and Si. The Si originated from the silicon substrate upon which the sample was deposited, whereas the O resulted from the small amount of oxidation that occurred. The crystalline structure of the products was characterised using X-ray powder diffraction (XRD), as shown in Fig. 1e; the products exhibited good crystallinity and were composed of the Fe_3_C (JCPDS No. 35-772) and Fe_5_C_2_ (JCPDS No. 36-1248) iron carbides. The XRD results suggested that the Fe_3_C iron carbide was predominant in the products. We thus concluded that the products consisted of 1D microfibres of Fe_3_C and Fe_5_C_2_ submicron spheres.

**Figure 3:**
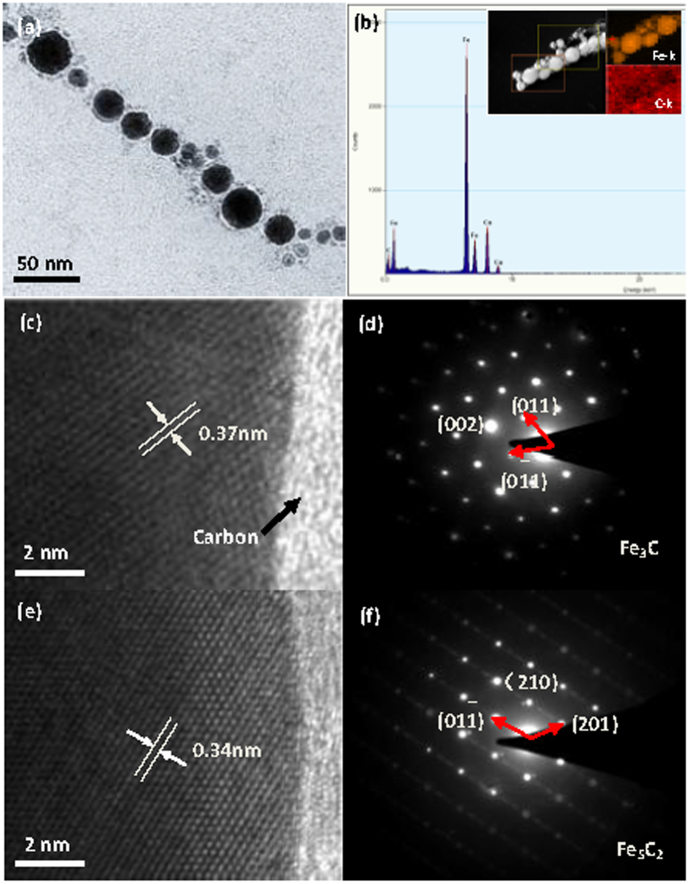
TEM bright-field image (a) of a single nanochain and the corresponding EDS (b), with inset STEM images.HRTEM images of two particles (c and d), and their corresponding SAED patterns (e and f).

The detailed structure of the microfibres was further revealed by the images shown in Fig. 3a–f, in which a single fibre exhibits both visible interparticle spacing between adjacent spheres (~10–30 nm) and the highly directed formation of a regular chain structure. The corresponding EDS pattern in Fig. 2b displays the elements Fe, C, and Cu. The Cu originated from the copper mesh on which the products were deposited. The inset depicts an STEM image of a single chain, revealing the corresponding distribution of Fe and C elements in the chain. Note that the carbon signal from the iron carbide was difficult to measure compared with that of the iron, and the presence of the double carbon layer on the copper grid decreased the accuracy in determining this distribution.

A high-resolution TEM (HRTEM) image (Fig. 3c) was taken of one nanosphere as shown in the inset of Fig. 3b, and the interplanar distance was measured at 0.37 nm; this result was in agreement with that for the (011) facet of the Fe_3_C iron carbide. The corresponding selected area electron diffraction (SAED) pattern (Fig. 3d) confirmed that the nanosphere was a single crystal. Based on careful analysis, the nanosphere was identified as orthorhombic iron carbide, which is indexed to the (011), 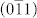, and (002) facets. Moreover, the HRTEM indicated that the nanosphere was covered with a layer of amorphous carbon, which may have reduced its level of oxidation. Another clear HRTEM image of the nanosphere is provided in Fig. 3e in which an interplanar distance of 0.34 nm was measured in accordance with the 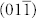 facet of Hägg iron carbide, Fe_5_C_2_. The corresponding SAED pattern (Fig. 3f) was indexed to the (201), (210), and 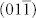 facets. The TEM results therefore confirmed that the micro-fibres were composed of submicron single-crystal iron carbide spheres (Fe_3_C and Fe_5_C_2_).

**Figure 4:**
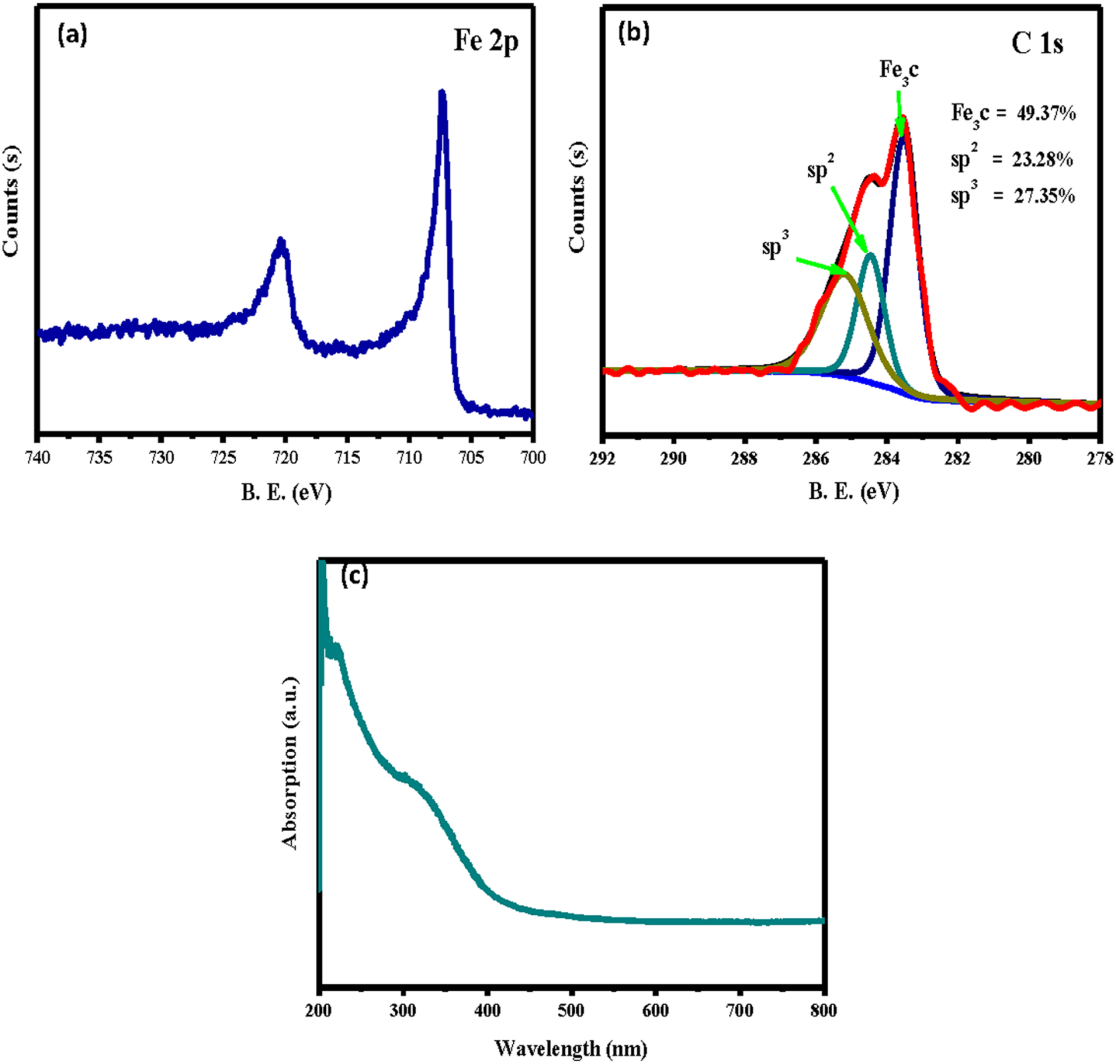
XPS spectra (a–b) and UV-vis absorption spectrum (c) for the products.

X-ray photoelectron spectroscopy (XPS) is a highly surface-sensitive technique for identifying the chemical state of materials; the XPS data are provided for the products in Fig. 4a–b. Because the iron carbide sphere surfaces were covered with amorphous carbon, we employed 3-kV Ar ion bombardment to remove the surface layer to a depth of 50 nm. For the Fe 2p region, the two peaks at 707.45 and 720.28 eV suggested the presence of iron carbide components^20^,^21^. The C1s spectrum was fitted with three components. The peak centred at 283.57 eV was coincident with that of the Fe_3_C combination^22^, while the other two peaks centred at 284.65 and 285.4 eV corresponded to the sp^2^- and sp^3^-bonded carbon^23^. Curve fitting of the C 1s spectra was performed using a standard least-squares algorithm to yield an 80% Gaussian Lorentzian-Gaussian peak shape. The measured full widths at half maximum (FWHM) for the Fe_3_C, sp^2^, and sp^3^ were 0.98, 0.88, and 1.5 eV, respectively. The integrated areas of these peaks correspond to Fe_3_C, sp^2^, and sp^3^ contents of 49.37, 23.28, and 27.35%, respectively. A Shirley background was used in each fit, and the XPS data confirmed that the nanospheres consisted of iron carbide with far fewer Fe-O combinations resulting from the oxidation of iron carbide under LAL^19^.

A well-defined absorption band was observed at ~300–400 nm in the UV-vis spectra of the products depicted in Fig. 4c and was assigned to the Fe_3_C iron carbide^24^. An absorption edge was also observed at wavelengths below 300 nm and was likely due to amorphous carbon^25^. These results indicate that the as-assembled 1D microfibres were formed from submicron single-crystalline iron carbide spheres.

### Magnetisation characterisations of the 1D microfibres

**Figure 5:**
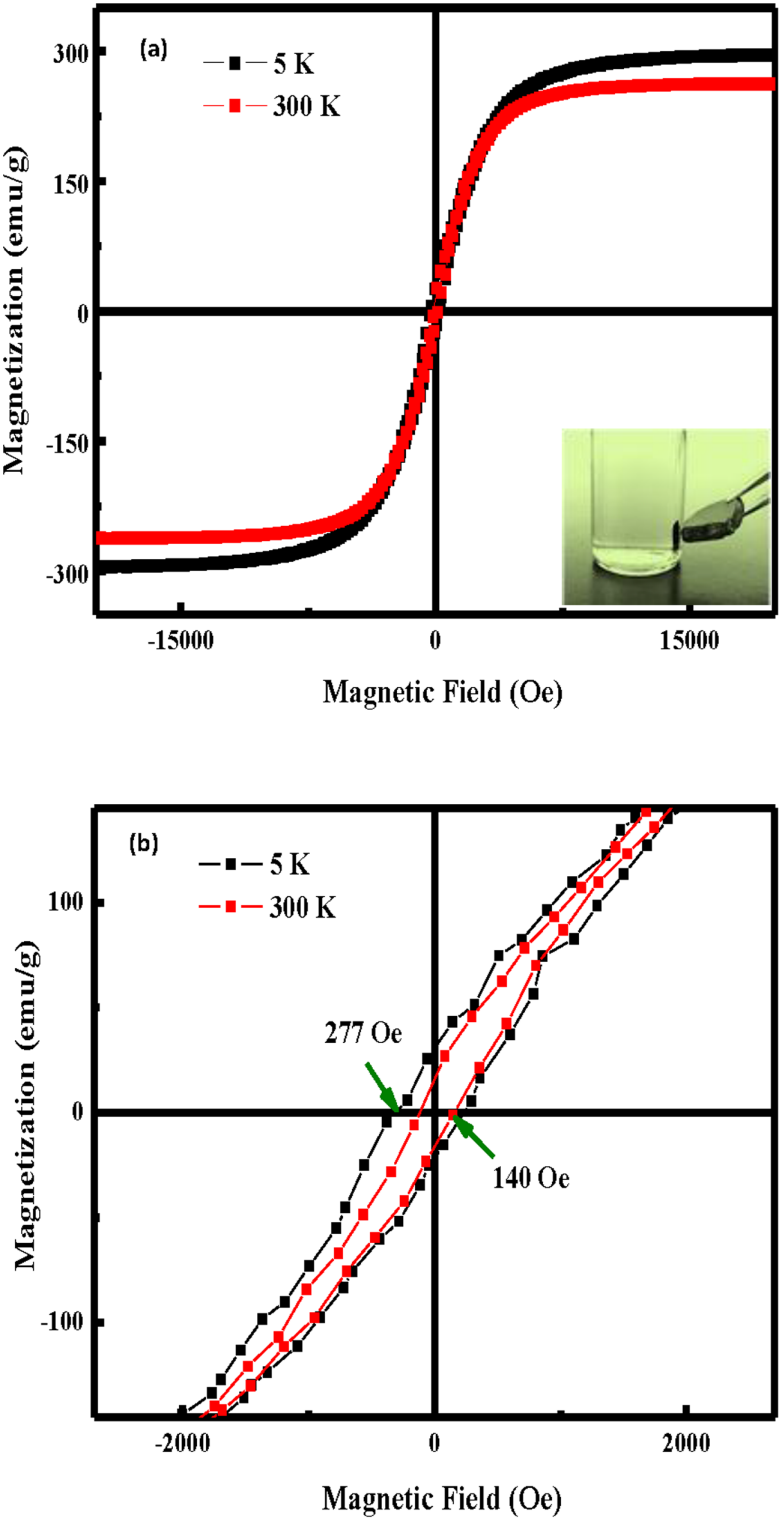
Magnetic properties of the products; the magnetic migration of the products toward a magnet occurred within a few seconds (see inset in a).

The field dependence of the magnetisation of the products was measured at room temperature (300 K) and at low temperature (5 K); the results are provided in Fig. 5, which reveals ferromagnetic behaviour in the products. The M-H plot in Fig. 5a indicates that the saturation magnetisation (M_s_) of the products was unexpectedly high, reaching 261 and 295 emu/g at 300 and 5 K, respectively. The remnant magnetisation (M_r_) was measured at 16 and 25 emu/g at 300 and 5 K, respectively, and the corresponding coercivity (H_c_) was measured at 140 and 277 Oe at 300 and 5 K, respectively, as indicated Fig. 5b. Surprisingly, the M_s_ value of the products was not only the best reported to date for iron nitride and carbide nanostructures^18^ but was also 5.5-times greater than the 47 emu/g for Fe_3_C nanoparticles^18^ and greater than the 212 emu/g for bulk Fe^26^. The magnetic behaviour of ordered MNP nanostructures is known to depend strongly on the interparticle interactions^27^,^28^. Generally, the spin disorder on the surface and the surface oxidation of the MNPs reduce the total magnetic moment^29^,^30^,^31^. However, in our case, the thin amorphous carbon layer on the surface of the nanospheres prevented the MNPs from oxidising. Similar reports have demonstrated M_s_ values above the bulk value^32^,^33^, which can be attributed to the special geometry of the magnetic nanostructures such as monopods and micro-octahedrons. Therefore, in our case, the abnormally giant saturation magnetisation of the MNP bundles was attributed to their unique structure and shape, whereas the exchange coupling between the nanospheres in the chains and that between the nanochains played crucial roles in enhancing the magnetic properties. The magnetic separability of the microfibres was tested in ethanol by placing a magnet near the glass bottle (see inset in Fig. 5a).

## Discussion

We now discuss the formation of the MNP microfibre under MF-LAL. The detailed mechanism for the nucleation and growth of NPs under LAL has been described previously^18^,^34^. When a strong magnetic field is applied during LAL, the process becomes more complicated due to the motion of the charged particles induced by the Lorentz force. Previous reports have demonstrated changes in the plume structure and dynamics with enhanced emission, ionisation, and velocity in the presence of a magnetic field^35^,^36^,^37^,^38^. Thus, we hypothesise that the magnetic field affects the nanoparticle formation. Based on the aforementioned results, a strong magnetic field plays a critical role during the formation of the 1D MNP chains. The MNPs dispersed in a solution typically experience two attractive forces: the van der Waals (vdW) force F_vdW_ and dipolar interactions F_dd_^38^,^39^. The vdW force originates from the electromagnetic fluctuations produced by the constant movement of positive and negative charges within all atoms, molecules, and bulk materials^40^, whereas the dipolar interactions originate from two magnetic particles. When an external magnetic field is applied, the dipole-field force F_m_ originating from ∇(**m**·**H**) should be considered because it can alter the energy distribution of the system^40^.

**Figure 6:**
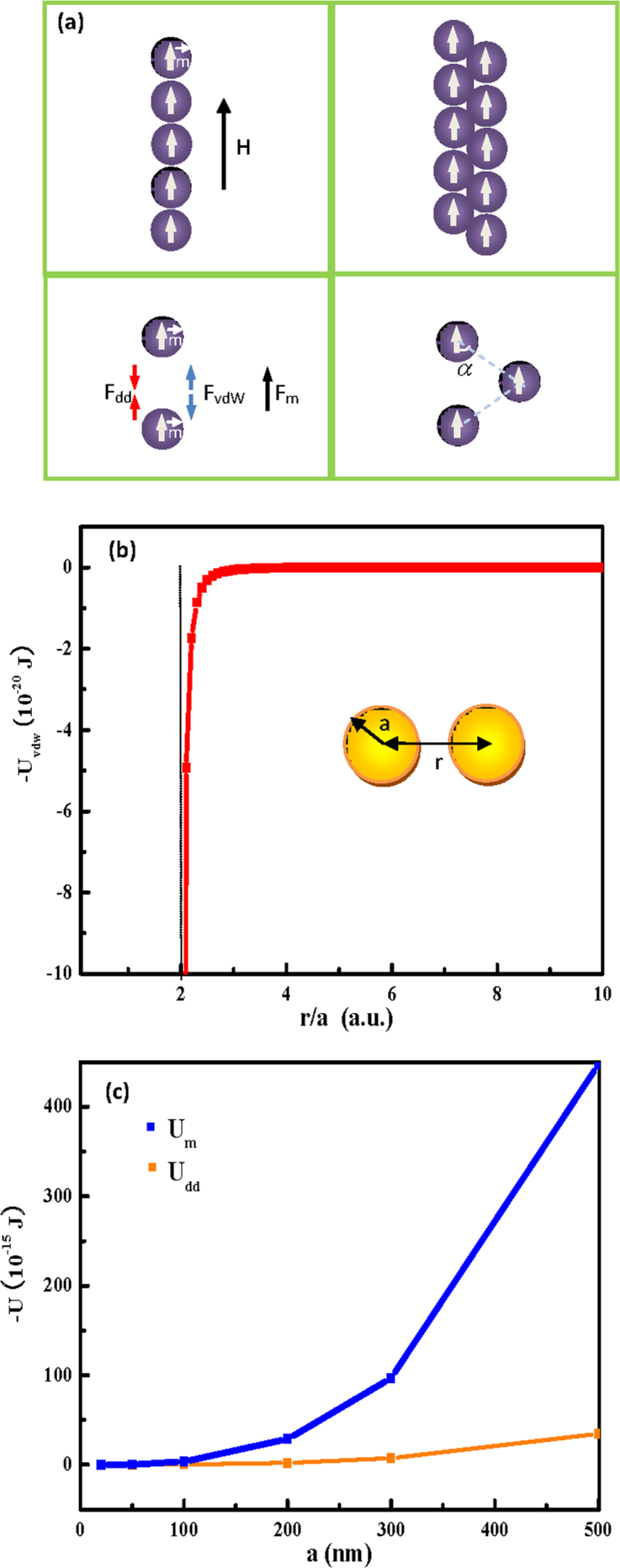
A physical model illustration (a) of a 1D chain, and an analysis of the forces between two nanospheres under the applied field. A plot of the vdW potential against r/a (b) and the dipolar-to-dipolar energy U_dd_, and the magnetostatic energy of particles with different sizes (c).

We calculated the three aforementioned interaction energies by considering a single linear chain system under a 9-T applied magnetic field (Fig. 6a). The idea was based on two basic assumptions: the MNPs were of the same size and were ordered one-by-one in a line along the magnetic field direction, and the nanospheres were magnetised in a spatially homogeneous fashion with a magnetic moment **m**. Thus, we can consider three interaction energies: the vdW potential U_vdW_, the magnetic dipole-dipole energy (U_dd_), and the magnetostatic energy U_m_.

The isotropous vdW interaction was estimated using the Hamaker integral approximation according to the following formula for two spheres of radius *a* separated by a centre-to-centre distance r^39^: 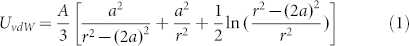
in which A is the Hamaker coefficient for ferrite particles (with a value of 10^−19^ J)^38^,^41^. The vdW interaction depended on the particle size and the distance between two particles, as indicated in Fig. 6b, and decreased sharply when r/a slightly exceeded 2. To simplify our experimental data, we set r/a = 2.1 in the following discussion. The characteristic *U_vdw_* for an arbitrary NP with r/a = 2.1 is a constant equal to 4.93 × 10^−20^ J.

The *U_dd_* represents the work required to bring two arbitrary dipoles with moments **m_1_** and **m_2_** from infinity to a finite separation of 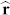
39: 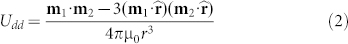
for a magnetic sphere with a radius a, a particle volume 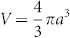 and a magnetic moment **m** = μ_0_*V***M** in which μ_0_ is the permeability of vacuum and **M** is the saturation magnetisation equated with that of the bulk material, M_s_^bulk^. For two identical dipoles in a line, as in our case (Fig. 6a), 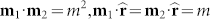, the interaction is attractive with a magnitude of 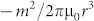 or 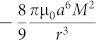. For iron carbide, the M_s_^bulk^ value is 140 emu/g^18^ or 8.54 × 10^5^ A/m, and the characteristic *U_dd_* at room temperature for a 100-nm NP is −2.76 × 10^−16^ J.

The magnetostatic energy of a dipolar particle in an external field **H** is given by *U_m_* = −**m**·**H**^39^. In our experiment, H = 9 T; thus, the *U_m_* of a 100-nm MNP was estimated at −3.58 × 10^−15^ J. For a 100-nm NP, *U_m_* is ~10^5^ and *U_dd_* is ~10^4^-times larger than *U*_vdw_. Note that these two interaction energies *U_dd_* and *U_m_* are strongest when **m** is the along the magnetic field direction, and they scale linearly with the particle volume such that the interactions between the particles are too weak to induce self-assembly. The *U_dd_* and *U_m_* values for 20-, 50-, 100-, 200-, 300-, and 500-nm particles are provided in Fig. 6c and have magnitudes of ~10^−18^–10^−14^ and ~10^−17^–10^−13^, respectively. For particles larger than 100 nm, the magnetostatic energy dominates the vdW interactions (~10^5^), resulting in a facile self-assembly of the 1D MNP chains. For particles smaller than 20 nm, under a uniform 9-T magnetic field, the magnetostatic energy is 1000-times greater than the vdW potential. Even if the magnetic field is removed, the *U_dd_* is still exceeds the *U_vdW_*; therefore, the chain structure is easily preserved even when the magnetic field is removed. In our experiments, the nanospheres larger than 100 nm were obviously connected—or tended to align—along the magnetic lines of force, favouring the formation of linear chains. The *U_dd_* is anisotropic, changes with the relative position of the two dipoles and is written as *m*^2^(1 − 3cos^2^*α*)/r^3^ in which *α* ranges from 0° to 90°. At the critical angle of 54.09°, *U_dd_* approaches zero. When 0 ≤ *α* < 54.09°, the interaction is attractive, but is repulsive for 54.09° < *α* ≤ 90°. This dipole-dipole interaction maintains the force balance for the nanospheres, thus allowing the formation of the microfibres depicted in Fig. 6a. In our case, the average particle size ranged from 200–300 nm, which is larger than that typically reported for LAL^19^. We hypothesised that the laser-induced melting mechanism proposed by Koshizaki plays an important role in fabricating a magnetic nanochain via MF-LAL^42^,^43^,^44^. Throughout the laser ablation process, the laser irradiation induced melting of the agglomerated NPs into submicron spheres. In our case, the fluence values used in the MF-LAL were similar to those used in the laser irradiation of colloids^42^,^43^,^44^.

The previously described calculations provide a general understanding of the MNP microfibre formation. First, the strong magnetic field magnetises the nanospheres produced via LAL. However, the magnetostatic energy is at least one order of magnitude greater than the thermal energy (k_B_T in which k_B_ is Boltzmann's constant and T is the absolute temperature; e.g., for 300 K, k_B_T is 4.14 × 10^−21^ J)^34^ and can overcome the thermal fluctuations to guide the particle movement. The nanospheres can then attract each other and tend to form linear chains along the line of magnetic force due to the head-to-tail alignment of the dipoles. Because the dipolar interaction and magnetostatic energies are considerably greater than the repulsive vdW potential, the 1D nanochains can be assembled.

**Figure 7:**
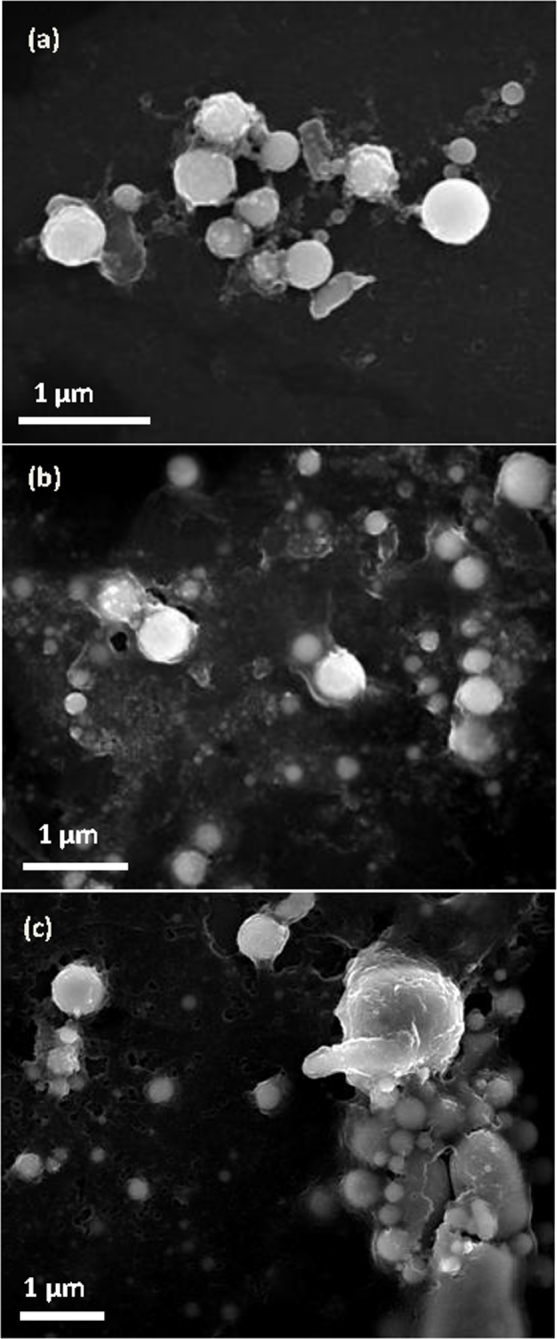
SEM images of laser ablation iron target in ethanol without the magnetic field. (a) the as-synthesized nanoparticles. The same sample placed in the magnetic field of 9 T after ablation at different time intervals: 2 hours (b) and 20 hours (c), respectively.

The given the calculations indicate that the applied magnetic field plays a crucial role in assembling the MNP chain. To confirm this role, a control experiment was conducted in which the magnetic field is present only after the synthesis and at various intervals. The submicron particles synthesised via LAL without any magnetic field but with all other conditions held constant are depicted in Fig. 7a. These nanoparticles are clearly disordered with no observable chains. Furthermore, the samples were firstly kept at room temperature for 2 and 20 hours, respectively, and then placed under magnetic field of 9 T. The corresponding SEM images of the samples provided in Fig. 7b and c display dispersed and random particles with no chains. Thus, we hypothesise that the magnetic field must be *in situ* during the laser ablation process to assemble the magnetic chains. In fact, our investigations indicated that the effects of the applied magnetic field on the chain fabrication via LAL and on the laser irradiation of colloids differ because LAL actually consists of two processes: laser ablation followed by the laser irradiation of the colloids. Therefore, our results demonstrated that ordered 1D chain bundles can be fabricated when a magnetic field is applied *in situ* during LAL. However, some disordered short chains form when the magnetic field is applied during the laser irradiation of the colloids.

**Figure 8:**
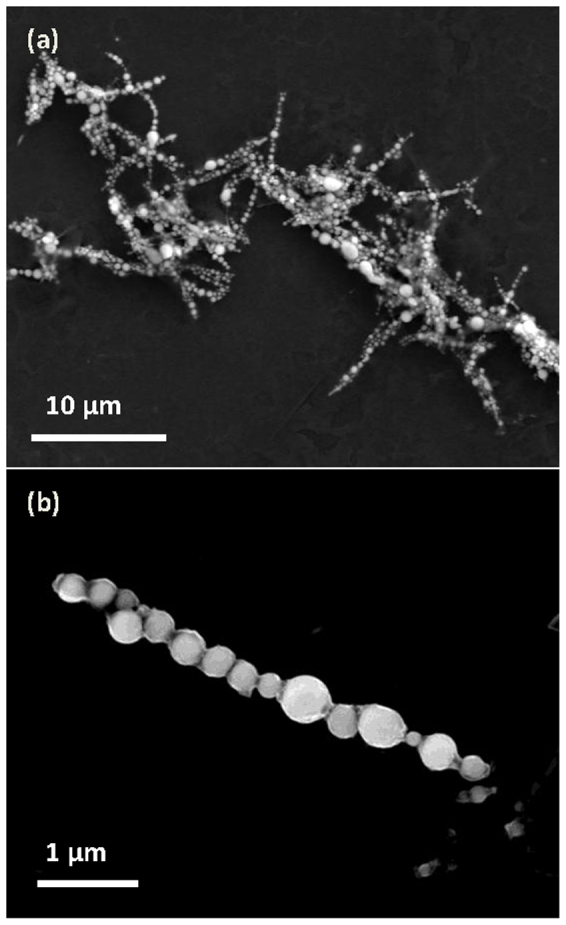
SEM images of the cobalt-based magnetic chains (a) fabricated by MF-LAL, and (b) an individual chain.

Amendola *et al.* reported the laser ablation of an iron target in different organic liquids for the synthesis of nanoparticles including iron carbide^24^. However, they did not characterise any magnetic properties of the as-synthesised nanoparticles. Additionally, Jakobi *et al.* reported the synthesis of magnetic Sm-Co and Ni-Fe alloys using LAL and the subsequent fabrication of short and disordered chains via polymerisation of the a nanoparticle/epoxy resin composite synthesised in an external magnetic field^45^. This two-step process involved the synthesis of magnetic nanoparticles via LAL without a magnetic field followed by the fabrication of short and disordered chains via polymerisation in an external magnetic field. Accordingly, we have developed a new one-step method involving magnetic field-assisted laser ablation in liquid (MF-LAL) to assemble 1D iron carbide microfibres with giant magnetisation. This novel technique allows researchers to choose and design interesting solid targets for fabricating MNP microfibres. For example, we used cobalt as a solid target and ethanol as a liquid to assemble cobalt-based magnetic chains via MF-LAL as shown in Fig. 8, which implies that MF-LAL could become a universal technique for the assembly of magnetic chains and chain-like structures. In fact, the fabrication of 1D chains during MF-LAL is a relatively complex process with physical mechanisms that vary with the conditions. For example, during laser ablation, nanoparticles sometimes form inside a cavitation bubble^46^. Therefore, in our case, some fabricated fibres may form inside the bubble or during its collapse.

**Figure 9:**
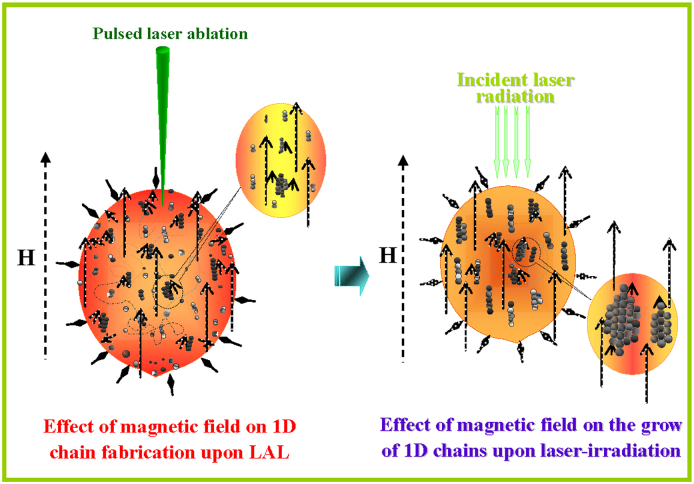
Schematic illustration of 1D chain fabrication upon MF-LAL.

Based on the previous discussions, the effects of the applied magnetic field on the fabrication of 1D chains via MF-LAL can be summarised as follows: In the first stage of laser ablation, the applied magnetic field not only induces the 1D chain formation of magnetic NPs but also forces these 1D chains to form a bundle. Then, the applied magnetic field can enhance the growth of the 1D chain bundles. A schematic illustration of the 1D chain fabrication via MF-LAL is provided in Fig. 9.

In summary, iron carbide microfibres were assembled via a simple, rapid, green, and relatively inexpensive route. These magnetic, ordered micro-fibres were constructed from 1D chains of submicron iron carbide spheres. Their saturation magnetisation was found to be 261 emu/g at room temperature, the best value reported to date for iron nitride and carbide nanoparticles and greater than that of bulk Fe. These findings make this unique magnetic nanostructure promising for a variety of biomedical and electronic applications.

## Methods

### Assembly of the magnetic microfibres

Laser ablation was performed using a Q-switch Nd:YAG laser device (Quanta-Ray Pro-250-, Newport Corporation, Spectra-Physics Div, Mountain View, CA 94043, U.S.A.) with a wavelength of 532 nm, a pulse width of 10 ns, a repeating frequency of 5 Hz, and a pulse energy of 100 mJ/pulse (a schematic illustration of the magnetic field-assisted LAL is provided in the Supplementary Information). The solid iron target (99.99% purity) used as the starting material was initially attached to the bottom of the glass chamber. Ethanol solvent (>99.7% pure) was slowly poured into the chamber until the target was submerged to a depth of ~5–8 mm. The container was then placed at the centre of the magnetic field, and the laser was focused onto the surface of the iron target with a focal length of 300 mm. A steady, strong, and uniform 9-T magnetic field was then applied, and the laser ablation of the iron target in the strong magnetic field was performed. The interaction lasted 90 min, and the solvent was collected for further measurements.

### Characterising the structural and magnetic properties

Scanning electron microscopy (SEM) images were obtained using a JSM-7600F field emission scanning electron microscope operated at 15 kV equipped with an EDS. XRD was performed using a D8 Advance X-ray diffractometer with Cu Kα radiation (λ = 1.54056 Å, 40 kV, 30 mA), and transmission electron microscopy (TEM) was conducted using an FEI Tecnai G2 F30 instrument at an accelerating voltage of 300 kV. These techniques were used to identify the crystal structure and morphology of the products. The composition of the particle surface was analysed via X-ray photoelectron spectroscopy (XPS) (ESCA Lab250), and UV-vis spectroscopy was carried out with a UV 3150 spectrophotometer (Shimadzu, Japan). The magnetic properties of the samples were measured on a Quantum Design PPMS-9T SQUID magnetometer.

## Author Contributions

G.W.Y. designed the experiments; Y.L. carried out the experiments and calculations and data analysis; P.L., J.X., H.B.L. assisted with some of the experiments; C.X.W. and G.W.Y. guided the work and the analysis. Y.L. and G.W.Y. wrote the paper.
